# Multiple time scales in modeling the incidence of infections acquired in intensive care units

**DOI:** 10.1186/s12874-016-0199-y

**Published:** 2016-09-01

**Authors:** Martin Wolkewitz, Ben S. Cooper, Mercedes Palomar-Martinez, Francisco Alvarez-Lerma, Pedro Olaechea-Astigarraga, Adrian G. Barnett, Martin Schumacher

**Affiliations:** 1 Institute for Medical Biometry and Statistics, Faculty of Medicine and Medical Center - University of Freiburg, Freiburg, Germany; 2 Freiburg Center of Data Analysis and Modelling, Albert-Ludwigs University Freiburg, Freiburg, Germany; 3 Mahidol-Oxford Tropical Medicine Research Unit, Faculty of Tropical Medicine, Mahidol University, Bangkok, 10400 Thailand; 4 Centre for Tropical Medicine and Global Health, Nuffield Department of Clinical Medicine, University of Oxford, Oxford, UK; 5 Hospital Universitari Arnau de Vilanova, Lleida, Universitat Autónoma de Barcelona, Barcelona, Spain; 6 Service of Intensive Care Medicine, Parc de Salut Mar, Barcelona, Spain; 7 Service of Intensive Care Medicine, Hospital de Galdakao-Usansolo, Bizkaia, Spain; 8 Institute of Health and Biomedical Innovation and School of Public Health and Social Work, Queensland University of Technology, Brisbane QLD, 4059 Australia

**Keywords:** Real-time approach, Left-truncation, Competing events, Incidence density, Infection

## Abstract

**Background:**

When patients are admitted to an intensive care unit (ICU) their risk of getting an infection will be highly depend on the length of stay at-risk in the ICU. In addition, risk of infection is likely to vary over calendar time as a result of fluctuations in the prevalence of the pathogen on the ward. Hence risk of infection is expected to depend on two time scales (time in ICU and calendar time) as well as competing events (discharge or death) and their spatial location. The purpose of this paper is to develop and apply appropriate statistical models for the risk of ICU-acquired infection accounting for multiple time scales, competing risks and the spatial clustering of the data.

**Methods:**

A multi-center data base from a Spanish surveillance network was used to study the occurrence of an infection due to Methicillin-resistant *Staphylococcus aureus* (MRSA). The analysis included 84,843 patient admissions between January 2006 and December 2011 from 81 ICUs. Stratified Cox models were used to study multiple time scales while accounting for spatial clustering of the data (patients within ICUs) and for death or discharge as competing events for MRSA infection.

**Results:**

Both time scales, time in ICU and calendar time, are highly associated with the MRSA hazard rate and cumulative risk. When using only one basic time scale, the interpretation and magnitude of several patient-individual risk factors differed. Risk factors concerning the severity of illness were more pronounced when using only calendar time. These differences disappeared when using both time scales simultaneously.

**Conclusions:**

The time-dependent dynamics of infections is complex and should be studied with models allowing for multiple time scales. For patient individual risk-factors we recommend stratified Cox regression models for competing events with ICU time as the basic time scale and calendar time as a covariate. The inclusion of calendar time and stratification by ICU allow to indirectly account for ICU-level effects such as local outbreaks or prevention interventions.

**Electronic supplementary material:**

The online version of this article (doi:10.1186/s12874-016-0199-y) contains supplementary material, which is available to authorized users.

## Background

The individual patients’ time at-risk (the length of stay at-risk in ICU) is a key determinant for ICU-acquired infections [1]. For infections which are based on rather exogenous acquisition routes [2], the risk might also depend on calendar time due to local outbreaks or fluctuations in the prevalence of other infectious patients, contaminated health-care worker in the same ICU or prevention interventions on the ICU-level [3]. In other words, the use of calendar time (in combination with stratification by ICU-level) allows to indirectly account for these transmission-associated effects. Thus, there are two time scales to be addressed in a risk factor analysis of ICU-acquired infections.

In time-to-event analysis, one basic time scale has to be selected. In general, possible choices for the basic time scale may include age, time since enrollment in a study, calendar time, or time since an event such as disease diagnosis. In many cases there may be no single time scale that is clearly more appropriate than others. The choice of time scale is, however, crucial: it affects the interpretation of the model and how risks and rates are assumed to vary over time; and in some cases different choices can even lead to apparently contradictory results [4–6]. The time scale therefore has to be chosen with care and the choice taken into account when interpreting results.

In the Cox proportional hazards model, the specification of the basic time scale plays a major role since time effects are not explicitly modeled as they are absorbed into the unspecified baseline hazard [4]. Thus, the underlying time scale provides the most flexible way to control for time effects. For instance, if the primary interest is in how a factor (such as a drug treatment) affects mortality for a disease, then a time scale of age might be most appropriate if the mortality rate is highly age-dependent but relatively unaffected by time since diagnosis, while a time scale of time since diagnosis might be more appropriate if the converse is true.

Time since enrollment (or time on study) is one of the most frequently used time scales, though there has been considerable debate about whether this choice has always been the most appropriate [4–6]. The use of calendar time as the basic time scale for longitudinal observational data (the so-called real-time approach) treats the population of interest as a dynamic population rather than a closed cohort [7, 8]. An application is, for instance, the impact of environmental exposure on pregnancy outcomes [6].

For ICU-acquired infections caused by transmissible pathogens such as MRSA and Vancomycin-resistant *Enterococci*, calendar time is often a natural choice when studying the effect of interventions on the ICU-level [9]. This is because hazards of acquiring the infection are likely to vary over calendar time as a result of fluctuations in the prevalence of the pathogen on the ward [3]. These fluctuations are typically unobserved as they result from asymptomatic carriage, making direct adjustment for the ICU-level prevalence impossible.

In addition to patient-individual characteristics, the risk of acquiring a MRSA infection in an ICU might also depend on spatio-temporal factors, i.e., *when* (calendar time) and *where* (which ICU) a patient requires intensive care.

The choice of calendar time as the basic time scale also controls for time-varying factors acting on the ICU-level such as changes in medical management, hygiene practices, patterns of antibiotic usage, staffing levels, and seasonal factors [3, 9–11].

The occurrence of ICU-acquired infection also depends on a second time scale, the patients’ individual time at-risk (i.e. time since patient admission to the ICU), with longer stays creating more opportunity for infection. This ICU exposure time is one the most important determinants for ICU-acquired infections and is frequently used for studying patient individual risk-factors such as age, morbidity, patient-individual antibiotic treatment or invasive devices [12]. Here, we discuss these two time scales which are crucial for the incidence of ICU-acquired Methicillin-Resistant *Staphylococcus aureus* (MRSA) infections.

The patients’ individual time at-risk ends with the occurrence of MRSA infection, ICU discharge or death in ICU since after the two latter events the risk of ICU-acquired infection is zero. Therefore, ICU discharge and death in ICU are competing events for ICU-acquired MRSA infections which should be considered in a risk factor analysis [12–14]. Ignoring these competing events can easily lead to heavily biased risk estimates [15] and wrong conclusions about the impact of risk factors [16]. Due to the presence of competing events, there are two metrics (the rate and the risk metric) in a risk factor analysis [17, 18]. Thus, for a complete analysis, it is necessary to perform event-specific hazard rate analyses (for MRSA infection, discharge and death) as well as a summary analysis for the cumulative risk of MRSA infection [19]. Furthermore, to account for the patients’ environment or geographical space, multi-level techniques are necessary [13].

The major aim of this paper is to find an appropriate model to study the incidence of MRSA infections by accounting for multiple time scales, competing risks and the hierarchical nature of the data. To do this, we explore, compare and combine the aforementioned time scales in a real ICU data setting. We calculate hazard rates with respect to the corresponding time scale and perform analyses based on the stratified Cox proportional hazards model to study patient-individual risk factors in a competing-risk framework.

## Methods

### Spanish ICU data

We used a multi-center data base from the Spanish surveillance network HELICS-ENVIN (http://hws.vhebron.net/envin-helics/), embedded in the HELICS project (Hospitals in Europe Link for Infection Control through Surveillance) [20]. We included ICUs which contributed to the registry between January 2006 and December 2011 and we included only patients who stayed at least two days in an ICU due to the definition of hospital-acquired infections. We excluded ICUs which contributed fewer than 500 patient admissions to the cohort to ensure a sufficient amount of patient time at risk for each ICU. The study population contains 81 intensive care units with 84,843 admissions (693,180 admission-days).

### Statistical methods

In the Additional file 1 is a Lexis diagram [21] of individual patient data from one selected ICU over 100 days in calendar time. It demonstrates how the data depend on the two time scales. In the following, we compare the two time scales in several steps. For the ICU time scale, the time origin is the time of admission. For the calendar time scale, patient admissions entered the model with *staggered* or *delayed entry* with left-truncation occurring at the time of admission.

#### Overall hazard rates

We used a penalized likelihood approach [22] to estimate the overall hazard rates *λ*^*k*^(*t*) separately for each event *k*: for ICU-acquired MRSA infection (the event of interest), death and discharge without MRSA. The overall hazard rates depend both on ICU or calendar time. The variation of the overall hazards due to different ICUs was accounted by using a shared frailty model [22]. More formally, let *c* represent the *calendar time* and *c*_0_ the *truncation time*, i.e., the admission time in calendar time scale. Thus, for each competing event *k* (MRSA, death without MRSA, discharge without MRSA) and the *i*-th ICU, the event-specific hazard rate with a shared frailty term ${Z^{k}_{i}}$ is defined as

$$\text{ICU time: } \lambda^{k}(c-c_{0}|{Z^{k}_{i}})={Z^{k}_{i}} {\lambda_{0}^{k}}(c-c_{0}) $$

$$\text{calendar time: } \tilde{\lambda}^{k}(c|c_{0},\tilde{Z}^{k}_{i})=\tilde{Z}^{k}_{i} \tilde{\lambda}_{0}^{k}(c|c_{0}) $$

with baseline hazard ${\lambda _{0}^{k}}(.)$ (or $\tilde {\lambda }_{0}^{k}(.)$) for event *k*; the term |*c*^0^ denotes the left-truncation time. The frailty term ${Z^{k}_{i}}$ (or $\tilde {Z}^{k}_{i}$) is a random effect which varies across ICUs (patients within ICU share the same frailty) and is assumed to be Gamma distributed with shape parameter 1/*θ*_*k*_ and inverse scale parameter 1/*θ*_*k*_, thus E(*Z*^*k*^)=1 and Var(*Z*^*k*^)=*θ*_*k*_. Large values of *θ*_*k*_ signify a closer positive relationship between patients within ICU and greater heterogeneity across ICUs.

#### Patient-level risk factors

We used event-specific Cox proportional hazards models (rate metric) and a Fine & Gray model [23] (risk metric) to explore covariate effects of vector *X* (gender, age, type of diagnosis, antibiotic treatment 48 h before and/or after ICU admission, trauma, days in hospital before ICU admission, APACHE II (Acute Physiology And Chronic Health Evaluation) score) comparing results between models where the timescale was calendar time and ICU time. The assumption of proportional hazards was checked via the inspection of the Schoenfeld residuals [24]; note that proportionality due to the rate metric does not lead to proportionality due to the risk metric but even if proportionality is not fulfilled the hazard ratio has the meaningful interpretation of an time-averaged effect [25]. We then model both times together by including the second time scale as a covariate. We stratified for ICU in order to allow the hazard to be different across ICUs, and hence we did not use the frailty terms.

### Models with one time scale

*Model 1a: ICU time as basic time scale*

$$\lambda^{k}(c-c^{0}|X)=\lambda_{0i}^{k}\left(c-c^{0}\right)\text{exp}\left(\sum_{j} {\beta^{k}_{j}} X_{j}\right) $$

This is an event-specific Cox model with ICU time as the basic time scale. The exponential of the regression coefficients ${\beta ^{k}_{j}}$ are corresponding hazard ratios of variable *X*_*j*_ and event *k*.

*Model 2a: calendar time as basic time scale (the real-time approach)*

$$\tilde{\lambda}^{k}(c|c^{0},X)=\tilde{\lambda}_{0i}^{k}(c|c^{0})\text{exp}\left(\sum_{j} \tilde{\beta}^{k}_{j} X_{j}\right) $$

This is an event-specific Cox model with calendar time as the basic time scale and *staggered* or *delayed entry* with left-truncation occurring at the time of admission.

*Model 3a: subdistribution for MRSA, basic time scale is ICU time*

In a competing risks setting, the cumulative incidence function for event *k* (CIF^*k*^(*t*)) depends on all event-specific hazards [26]. This can be seen with following formula with *t*=*c*−*c*^0^:

$$\begin{aligned} \text{CIF}^{k}(t)={\int_{0}^{t}} \left(\text{exp}\left(-\sum_{\text{all events }i} {\int_{0}^{u}} \lambda^{i} (v) dv \right)\right) \times \lambda^{k} (u) du \end{aligned} $$

We are basically interested in MRSA infection as our event of interest. In simple words, the formula above is the product of the time-dependent probability of staying alive at-risk on ICU and the conditional probability of acquiring a MRSA infection. Note that the probability of staying alive at-risk on ICU depends on the competing events hazards (discharge or dying without MRSA) in addition to the MRSA hazard.

Fine & Gray [23] defined the subdistribution hazard which is in our setting the probability of the MRSA infection given that a patient has stayed in ICU up to time t without a MRSA infection or has had the competing event (death ore discharge) prior to time t [27]. Thus, the risk sets for the subdistribution hazard are unnatural (discharged and died patients remain technically at-risk). However, unlike the event-specific hazard, the subdistribution hazard is directly linked to the corresponding cumulative incidence function of MRSA infection. Based on the subdistribution hazard, Fine & Gray proposed a proportional hazards model to study risk factors on the risk metric. The resulting subdistribution hazard ratios of an exposure can be interpreted as effects which can be seen when plotting cumulative incidence functions, grouped by exposure categories.

### Models with two time scales

*Model 1b: model 1a plus year of admission as a covariate*

$$\lambda^{k}(c-c^{0}|X)=\lambda_{0i}^{k}\left(c-c^{0}\right)\text{exp}\left(\sum_{j} {\beta^{k}_{j}} X_{j}+\gamma f\left(c^{0}\right)\right) $$

with *f*(*c*^0^) as the calendar year of admission. If necessary, other more detailed functions (which, for instance, includes also the calendar month of admission) can be chosen.

*Model 2b: model 2a plus length of stay at-risk as a time-dependent covariate*

$$\tilde{\lambda}^{k}(c|c^{0},X)=\tilde{\lambda}_{0i}^{k}\left(c|c^{0}\right)\text{exp}\left(\sum_{j} \tilde{\beta}^{k}_{j} X_{j}+\gamma g\left(c-c^{0}\right)\right) $$

with *g*(*c*−*c*^0^) as a function to categorize ICU time in 0-4, 5-9, 10-14 and 15+ days. If necessary, other categorizations can be chosen.

*Model 3b: model 3a plus year of admission as covariate*.

## Results

### Overall hazards

**Fig. 1:**
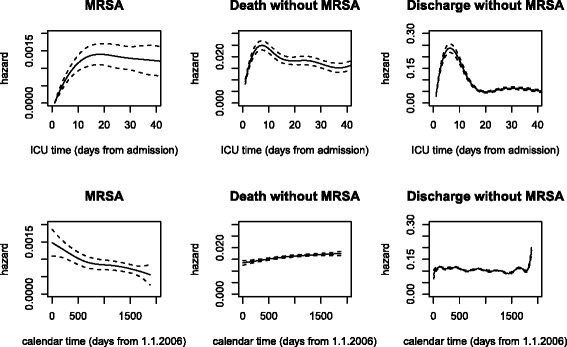
Event-specific hazard rates depending on ICU time (*upper panels*) or calendar time (*lower panels*)

The overall hazard rates are displayed in Fig. 1. The bottom row is clearly showing what is happening (with respect to event risk) over a long time period (perhaps reflecting more what is going on at the ICU level) whereas the top row is showing what is happening at the patient level in the short term since admission to an ICU. With the ICU time scale, the hazard of MRSA acquisition increases from 0 to 0.0015 within the first 15 days from admission (perhaps due to the fact that an MRSA infection often follows a MRSA colonisation which itself also requires some exposure time in ICU). After 15 days, the MRSA hazard slightly decreases. The hazard reflects the (unadjusted) instantaneous risk for an ICU patient to acquire a ICU-acquired MRSA infection during his or her ICU stay. This hazard has to be interpreted jointly with the corresponding hazards of the competing events since the cumulative risk of MRSA infection depends also on the discharge and death hazards. The hazards of death or discharge without MRSA are also increasing within the first week after admission (see Fig. 1: up to 0.025 at day 7 for death and up to 0.25 at day 7 for discharge). After 10-15 days, the hazard of death without MRSA is about 0.02 and the hazard of discharge without MRSA is much lower (about 0.05) in contrast to earlier days.

The MRSA hazard rate with respect to calendar time shows how the hazard steadily decreases from the January 2006 to December 2010 on the ICU level (Fig. 1 bottom left), potentially due to the implementation of prevention strategies [28]. During the years 2006–2010, the death hazard rate without MRSA slightly increased from 0.014 to 0.017. The discharge hazard rate remains more or less constant (about 0.1) over calendar time. Details regarding the random effects of ICU are displayed in the Additional file 1.

### Effects of patient individual risk-factors

The covariates of interest are listed in Table 1. Descriptive statistics indicate the following variables are associated with calendar time. The mean number of days in hospital before ICU admission steadily increased from about 9.5 days in year 2006 up to 18.5 days in year 2010+. The proportion of patient admissions with trauma steadily decreased from 11.5 % in 2006 to 7.1 % in 2010+. The proportion of admissions receiving antibiotic treatment 48 h before and/or after ICU admission steadily decreased from about 30 to 19 %.

**Table 1:** Description of study population

General	Frequency (%)
Number of admissions	84843
Number of admission-days	693180
Number of ICUs	81
Number of MRSA infections during ICU stay	469
Number of deaths without MRSA infections during ICU stay	11131
Number of discharges without MRSA infections from ICU	72701
Number of administrative censored admissions	542
Overall risk of MRSA infections (censored excluded)	0.56 %
Overall rate of MRSA infections	0.068 %
Calendar year of admission	
2006 (reference)	11301 (13.32)
2007	14862 (17.52)
2008	17654 (20.81)
2009	19361 (22.82)
2010+	21665 (25.54)
Parient level covariates	
APACHE II score 0-10 (reference)	30291 (35.70)
APACHE II score 11-20	35428 (41.76)
APACHE II score 21-30	15187 (17.90)
APACHE II score >30	3937 (4.64)
Age (years) 0-40	42232 (49.78)
Age (years) 40-60	10227 (12.05)
Age (years) 61-80 (reference)	22989 (27.10)
Age (years)>80	9395 (11.07)
Days in hospital before ICU admission:	
0-3 (reference)	67301 (79.32)
4-6	4616 (5.44)
6-10	3950 (4.66)
>10	8976 (10.58)
Type of diagnosis:	
Cardiovascular (reference)	41990 (49.49)
Respiratory	11816 (13.93)
Gastrointestinal	10958 (12.92)
Central nervous system	14473 (17.06)
Other diagnoses	5606 (6.61)
Antibiotic treatment 48 h before and/or after ICU admission	18052 (21.28)
Gender (male)	55308 (65.19)
Origin: community (reference)	41640 (49.08)
Origin: hospital/ICU	43203 (50.92)
Trauma	7167 (8.45)

#### One time scale

In Table 2, the effects of several risk factors are listed from Models 1a and 2a. The hazard ratios do not significantly differ much (estimates for one model are generally within the 95 % confidence interval of the corresponding estimates in the other model) but they have different interpretations. If ICU time is used as the time scale, then the effects of interest are directly adjusted for ICU time effects (such as the patient-individual intensity of the underlying morbidity – generally the more critical care is needed the longer the ICU stay). By using calendar time, the effects of interest are directly adjusted for calendar time effects such as local outbreaks, changing hygiene practices or intervention strategies. Thus, the combination of using the calendar time scale and the additional stratification by ICU addresses the spatio-temporal dynamics of ICU-acquired MRSA infections. That means that the risk factors are studied beyond this spatio-temporal pattern.

**Table 2:** Results from multivariate analysis (one time scale). Event-specific regression analyses based on Cox proportional regression models as described in main text. Proportionality could be assumed after the inspection of Schoenfelds’ residuals. Event-specific hazard ratios are displayed with 95 %-confidence interval in brackets

Risk factors	MODEL 1a basic time scale: ICU time			MODEL 2a basic time scale: calendar time		
	MRSA	Death without MRSA	Discharge without MRSA	MRSA	Death without MRSA	Discharge without MRSA
Patient level covariates						
APACHE II score 11–20 vs. 0–10	1.35 (1.00–1.83)	2.05 (1.90–2.23)	0.62 (0.61–0.63)	1.78 (1.30–2.44)	2.20 (2.03–2.39)	0.63 (0.62–0.65)
APACHE II score 21–30 vs. 0–10	1.50 (1.09–2.07)	4.23 (3.91–4.57)	0.37 (0.36–0.38)	2.22 (1.59–3.12)	4.76 (4.39–5.16)	0.37 (0.31–0.38)
APACHE II score>31 vs. 0–10	1.50 (0.99–2.28)	6.86 (6.30–7.47)	0.24 (0.23–0.26)	2.75 (1.76–4.32)	8.02 (7.33–8.78)	0.24 (0.22–0.25)
Age (years) 0–40 vs. 61–80	1.09 (0.80–1.45)	0.62 (0.57–0.67)	1.08 (1.05–1.10)	1.03 (0.75–1.41)	0.62 (0.57–0.85)	1.06 (1.03–1.09)
Age (years) 40–60 vs. 61–80	0.96 (0.77 –1.20)	0.81 (0.77–0.85)	0.99 (0.98–1.01)	0.98 (0.77–1.24)	0.81 (0.77–0.85)	0.99 (0.97–1.01)
Age (years)>80 vs. 61–80	0.72 (0.47–1.11)	1.61 (1.52–1.70)	1.12 (1.09–1.15)	0.63 (0.40–0.98)	1.62 (1.53–1.73)	1.15 (1.12–1.19)
Days in hospital before ICU admission:						
4-6 vs. 0–3	1.14 (0.78–1.66)	1.11 (1.03–1.20)	0.89 (0.86–0.92)	1.31 (0.87–1.96)	1.12 (1.03–1.22)	0.91 (0.88–0.95)
6-10 vs. 0–3	1.16 (0.78–1.73)	1.22 (1.13–1.32)	0.89 (0.85–0.92)	1.10 (0.71–1.70)	1.23 (1.12–1.34)	0.91 (0.88–0.95)
> 10 vs. 0–3	1.09 (0.8–1.46)	1.24 (1.17-1.31)	0.84 (0.81–0.86)	1.21 (0.89–1.67)	1.24 (1.16–1.32)	0.85 (0.82–0.87)
Type of diagnosis:						
Respiratory vs. cardiovascular	1.10 (0.84–1.45)	0.98 (0.92–1.39)	0.75 (0.73–0.77)	1.42 (1.06–1.90)	0.99 (0.93–1.05)	0.75 (0.74–0.77)
Gastrointestinal vs. cardiovascular	1.29 (0.95–1.74)	1.01 (0.95–1.07)	0.84 (0.82–0.86)	1.48 (1.07–2.04)	1.01 (0.95–1.08)	0.82 (0.80–0.84)
Central nervous system vs. cardiovascular	1.18 (0.90–1.55)	1.37 (1.30–1.45)	0.77 (0.75–0.78)	1.39 (1.04–1.87)	1.41 (1.32–1.49)	0.75 (0.73–0.77)
Other diagnoses vs. cardiovascular	0.92 (0.62–1.36)	0.84 (0.76–0.91)	0.86 (0.84–0.89)	0.95 (0.58–1.56)	0.84 (0.76–0.94)	0.99 (0.93–1.02)
Antibiotic treatment 48 h before and/or after ICU admission	1.21 (0.97–1.51)	1.09 (1.04–1.14)	0.77 (0.76–0.79)	1.44 (1.12–1.86)	1.13 (1.07–1.19)	0.72 (0.70–0.73)
Gender	1.14 (0.93–1.39)	1.01 (0.97–1.05)	0.99 (0.96–1.01)	1.22 (0.99–1.52)	0.99 (0.96–1.01)	0.98 (0.91–0.99)
Origin (hospital/ICU vs. community)	0.93 (0.74–1.19)	0.95 (0.91–1.00)	1.03 (1.01–1.04)	0.95 (0.75–1.22)	0.96 (0.91–1.01)	1.03 (1.01–1.05)
Trauma	1.25 (0.94–1.68)	0.67 (0.62–0.73)	0.74 (0.72–0.76)	1.41 (1.03–1.94)	0.67 (0.61–0.73)	0.73 (0.70–0.75)

The estimated effects in Table 2 are different according to the time scale used. This is especially true for the APACHE II score, type of diagnosis, antibiotic treatment and trauma where the effects are more pronounced when calendar time is taken as the basic time scale. One explanation is that these factors are highly associated with the severity of patients’ illness and the ICU time captured already part of this severity.

#### Two time scales

If calendar year is introduced in the model as an additional covariate (Table 3), the results for the patient-individual factors barely change (comparing effects between models 1a and b) even though calendar year is associated with all outcomes, MRSA as well as the competing events death or discharge. This consistency might be due to the noncorrelation of calendar and ICU time. Comparing models 2a and b, one can see that including the covariate length of stay at-risk in the model changes the hazard ratios; they are now very similar to those from model using ICU time scale (model 1b). Table 3 shows the results of the two models 1b and 2b accounting for both time scales simultaneously. Both models yield very similar results.

**Table 3:** Results from multivariate analysis (two time scales). Event-specific regression analyses based on Cox proportional regression models as described in main text. Proportionality could be assumed after the inspection of Schoenfelds’ residuals. Eventspecific hazard ratios are displayed with 95 %-confidence interval in brackets

Risk factors	MODEL 1a basic time scale: ICU time			MODEL 2a basic time scale: calendar time		
	MRSA	Death without MRSA	Discharge without MRSA	MRSA	Death without MRSA	Discharge without MRSA
Patient level covariates						
APACHE II score 11–20 vs. 0–10	1.36 (1.01–1.83)	2.05 (1.90–2.21)	0.62 (0.61–0.63)	1.40 (1.02–1.93)	2.14 (1.97 2.32)	0.64 (0.63–0.65)
APACHE II score 21–30 vs. 0–10	1.52 (1.11–2.09)	4.22 (3.90–4.56)	0.37 (0.36–0.38)	1.53 (1.08–2.17)	4.58 (4.22 4.97)	0.38 (0.37–0.39)
APACHE II score>31 vs. 0–10	1.52 (1.00–2.32)	6.83 (6.28–7.44)	0.25 (0.23–0.26)	1.86 (1.18–2.96)	7.73 (7.05 8.45)	0.25 (0.24–0.26)
Age (years) 0-40 vs. 61–80	1.07 (0.80–1.44)	0.62 (0.57–0.67)	1.08 (1.05–1.10)	0.97 (0.71–1.34)	0.62 (0.57 0.67)	1.05 (1.02–1.08)
Age (years) 40-60 vs. 61–80	0.97 (0.78–1.21)	0.81 (0.77–0.85)	1.00 (1.00–1.01)	0.93 (0.74–1.18)	0.81 (0.77 0.85)	0.99 (0.97–1.01)
Age (years)>80 vs. 61–80	0.73 (0.47–1.11)	1.60 (1.52–1.70)	1.12 (1.09–1.15)	0.71 (0.45–1.11)	1.63 (1.53 1.74)	1.13 (1.10–1.16)
Days in hospital before ICU admission:						
4-6 vs. 0–3	1.15 (0.79–1.68)	1.11 (1.03–1.20)	0.89 (0.86–0.92)	1.30 (0.86–1.97)	1.12 (1.03 1.22)	0.92 (0.89–0.96)
6-10 vs. 0–3	1.16 (0.78–1.73)	1.22 (1.13–1.32)	0.89 (0.86–0.92)	1.00 (0.64–1.56)	1.22 (1.12 1.33)	0.91 (0.88–0.95)
> 10 vs. 0–3	1.10 (0.82–1.48)	1.24 (1.17–1.31)	0.84 (0.81–0.86)	1.21 (0.88–1.66)	1.23 (1.16 1.32)	0.86 (0.83–0.88)
Type of diagnosis:						
Respiratory vs. cardiovascular	1.13 (0.86–1.49)	0.97 (0.92–1.02)	0.76 (0.74–0.77)	1.30 (0.96–1.75)	0.97 (0.92 1.03)	0.77 (0.75–0.79)
Gastrointestinal vs. cardiovascular	1.28 (0.95–1.73)	1.01 (0.95–1.07)	0.84 (0.82–0.86)	1.39 (1.00–1.94)	1.01 (0.94 1.08)	0.84 (0.82–0.86)
Central nervous system vs. cardiovascular	1.18 (0.40–1.55)	1.37 (1.30–1.45)	0.77 (0.75–0.78)	1.32 (0.98–1.77)	1.39 (1.30 1.47)	0.77 (0.75–0.79)
Other diagnoses vs. cardiovascular	0.90 (0.60–1.33)	0.84 (0.76–0.92)	0.86 (0.84–0.89)	0.93 (0.56–1.54)	0.84 (0.76 0.94)	1.01 (0.98–1.05)
Antibiotic treatment 48 h before and/or after ICU admission	1.12 (0.89–1.40)	1.10 (1.05–1.15)	0.76 (0.75–0.78)	1.29 (0.99–1.67)	1.11 (1.06 1.17)	0.72 (0.71–0.74)
Gender	1.14 (0.94–1.40)	1.01 (0.97–1.05)	1.00 (1.00–1.01)	1.22 (0.98–1.52)	1.00 (0.96 1.04)	0.99 (0.97–1.00)
Origin (hospital/ICU vs. community)	0.94 (0.75–1.17)	0.95 (0.91–1.00)	1.03 (1.01–1.05)	0.95 (0.74–1.21)	0.96 (0.91 1.01)	1.02 (1.00–1.04)
Trauma	1.20 (0.40–1.61)	0.67 (0.62–0.73)	0.74 (0.72–0.76)	1.29 (0.94–1.78)	0.66 (0.60 0.72)	0.74 (0.71–0.76)
Second time scale						
Calendar year of admission						
2007 vs. 2006	0.68 (0.51–0.92)	1.05 (0.98–1.13)	0.95 (0.93–0.98)			
2008 vs. 2006	0.57 (0.43–0.78)	1.11 (1.04–1.20)	0.92 (0.90–0.94)			
2009 vs. 2006	0.59 (0.44–0.79)	1.11 (1.03–1.19)	0.88 (0.86–0.91)			
2010+ vs. 2006	0.49 (0.36–0.67)	1.15 (1.07–1.24)	0.94 (0.91–0.96)			
Length of stay						
5-9 vs. 0–4 days				4.63 (3.30–6.48)	1.35 (1.28 1.42)	1.47 (1.44–1.50)
10-14 vs. 0–4 days				4.66 (3.18–6.84)	1.29 (1.20 1.38)	0.85 (0.82–0.87)
>14 vs. 0–4 days				5.42 (3.87–7.57)	1.05 (0.99 1.11)	0.61 (0.59–0.63)

#### Subdistribution approach

**Fig. 2:**
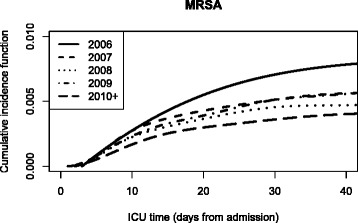
Cumulative incidence function of ICU-acquired MRSA depending on ICU time scale and stratified per calendar year on admission

Since most covariates are also associated with the competing events for MRSA, it is also required to study the covariate effects on the cumulative incidence function (CIF) [19, 23]. The CIF depends on the underlying time scale. The CIF is a very relevant quantity when the ICU time scale is used since it reflects how the individual patient risk accumulates with time spent in the ICU (Fig. 2). In contrast, displaying the cumulative incidence function with the calendar time scale is not useful. One way to account for calendar time is adjusting for the admission year as a covariate (model 3b). Table 4 shows how the covariates are associated with the subdistribution hazard of MRSA as our event of interest. The importance of competing events in such a risk factor analysis can be seen when comparing the results of model 3b with those from model 1b. Depending on the metric used (rate or risk), the effects can be very different. For instance, APACHE II score is moderately associated with an increased *hazard rate* of MRSA infection (e.g., HR=1.52 (95 %-CI: 1.00-2.32)) for APACHE II score >31 vs. 0-10) but highly associated with an increased *risk* of MRSA infection (subdistribution HR=5.79 (95 %-CI: 3.82-8.76)) for APACHE II score >31 vs. 0-10). This phenomena can be explained by considering the effects on the competing events: patients with higher APACHE II scores are associated with an increased death hazard (they die faster) but also with an decreased discharge hazard (they stay longer at ICU). Since most patients are discharged, the actual at-risk time is prolonged for patients with higher APACHE II scores. Therefore, more MRSA infections occur in this patient group since longer stays create more opportunity for infection. The difference between the two metrics can also go in the other direction: patients older than 80 years have a (non-significantly) lower hazard to acquire a MRSA infection than patients aged between 61-80 years (HR=0.73 (95 %-CI: 0.47-1.11)). However, the cumulative risk for MRSA infection is much lower (subdistribution HR=0.47 (95 %-CI: 0.31-0.71)) since the older patient group die faster without MRSA (HR=1.60 (95 %-CI:1.52-1.70)) and is discharged faster (HR=1.12 (95 %-CI: 1.09-1.15)) meaning that the at-risk time for MRSA infection is reduced.

**Table 4:** Results from multivariate analysis (subdistribution analysis). Regression analyses based on adapted Cox proportional regression model (Fine & Gray model) as described in main text. Proportionality could be assumed after the inspection of Schoenfelds’ residuals. Subdistribution hazard ratios are displayed with 95 %-confidence interval in brackets

Risk factors	MODEL 3a one time scale subdistribution	MODEL 3b two time scales subdistribution
	approach basic time scale: ICU time	approach basic time scale: ICU time
	subdistribution MRSA	
Patient level covariates		
APACHE II score 11–20 vs. 0–10	3.05 (2.27–4.11)	3.06 (2.27–4.13)
APACHE II score 21–30 vs. 0–10	4.92 (3.58–6.76)	4.96 (3.61–6.82)
APACHE II score>31 vs. 0–10	5.71 (3.77–8.64)	5.79 (3.82–8.76)
Age (years) 0–40 vs. 61–80	1.14 (0.86–1.53)	1.13 (0.85–1.51)
Age (years) 40–60 vs. 61–80	1.04 (0.83–1.29)	1.04 (0.84–1.30)
Age (years)>80 vs. 61–80	0.46 (0.30–0.71)	0.47 (0.31–0.71)
Days in hospital before ICU admission:		
4-6 vs. 0–3	1.32 (0.90–1.92)	1.32 (0.91–1.93)
6-10 vs. 0–3	1.44 (0.97–2.15)	1.44 (0.97–2.15)
> 10 vs. 0–3	1.32 (0.98–1.77)	1.32 (0.98–1.78)
Type of diagnosis:		
Respiratory vs. cardiovascular	1.69 (1.29–2.21)	1.71 (1.31–2.25)
Gastrointestinal vs. cardiovascular	1.77 (1.31–2.38)	1.76 (1.31–2.38)
Central nervous system vs. cardiovascular	1.59 (1.21–2.09)	1.60 (1.22–2.11)
Other diagnoses vs. cardiovascular	1.39 (0.96–2.02)	1.40 (0.96–2.03)
Antibiotic treatment 48 h before and/or after ICU admission	1.39 (1.11–1.73)	1.33 (1.06–1.66)
Gender	1.18 (0.96–1.44)	1.17 (0.96–1.43)
Origin (hospital/ICU vs. community)	0.95 (0.76–1.19)	0.96 (0.76–1.20)
Trauma	1.87 (1.34–2.49)	1.80 (1.35–2.41)
Calendar year of admission		
2007 vs. 2006		0.71 (0.53–0.96)
2008 vs. 2006		0.64 (0.47–0.86)
2009 vs. 2006		0.73 (0.55–0.97)
2010+ vs. 2006		0.52 (0.38–0.70)

The inclusion of the calendar year of admission in the subdistribution analysis has no impact on the patient-level covariates. Even though the effect of admission year is strong, the estimates from model 3a and 3b are almost the same.

## Discussion

In this paper, we compared two important time scales (ICU and calendar time) for modeling the incidence of ICU-acquired MRSA infections. Both time scales have a strong influence on the hazard rate and cumulative risk of MRSA infections in ICUs but also of the competing events (death and discharge). We showed that hazard ratios of patient individual risk-factors of MRSA infections can differ depending on the underlying time scale in Cox regression models. This difference can be overcome by using both time scales simultaneously.

One strength of this study was the application of advanced statistical methods on a large data base which was necessary to model calendar time and patient-individual characteristics simultaneously. We used competing risks models based on the semi-parametric Cox proportional hazards model and shared frailty models even though other parametric frailty models could be applied in such settings. In a recent review about analyses of hospital-acquired infection risk factors, Brown and colleagues also emphasized the need to adjust for the at-risk time and period effects (calendar time) [1]. In addition to the time scales, we gave emphasis on competing risks since the interpretation and conclusions of the results might be very different [18].

This study has limitations. First, the data have been collected from volunteer ICUs, thus data can be subject to reporting, information or selection bias. Second, part of the ICUs contributed the whole study period from January 2006 and December 2011 whereas others contributed only 3 months per year (April to June) and some ICUs started their continuous contribution at some time between 2006 and 2011. This might affect our findings, particular for calendar time. Third, it can be assumed that transmission dynamics play the main role in MRSA *colonisation* (which might later lead to MRSA *infection*). In our setting, we were not able to study MRSA colonisation rates because such an evaluation requires regular swabs from patients to detect asymptomatic carriage. Instead, we focused on MRSA infections because this is both the clinically important outcome and the outcome for which the best data are available. Fourth, we studied only time-independent risk factors in the subdistribution approach. Time-dependent risk factors (such as antibiotic treatment or invasive devices during ICU) can be introduced in the event-specific analyses but there are challenges in interpreting results from risk metric approaches in presence of time-dependent risk factors [18].

The choice of the basic time scale has been discussed in the statistical literature. Andersen and Keiding [29] stated that the underlying time scale should be chosen ’for which the variation in the hazard is unknown or is expected to be dramatic and a parametric description is less important’. According to Pencina et al. [30], one should ask the question: ’Which approach seems to better capture the nature of the data that are to be modeled, and which is more suitable to answer the research questions?’.

Given these general statements and based on our findings regarding MRSA infections, ICU time should be chosen as the basic time scale when studying patient individual risk-factors. To indirectly account for exogenous factors (such as local outbreaks or implementation of prevention strategies on the ICU-level), we recommend to use calendar time as a covariate in Cox regression models and stratify by ICU. A further advantage of this time scale choice is that the cumulative incidence functions of MRSA infections can be studied in a competing event framework.

## Conclusions

Risk factor analyses of general ICU-acquired infections are already complex due to the presence of competing risks during the time in ICU as well as unit-level effects. Moreover, the analysis of ICU-acquired infections requires the involvement of calendar time. By accounting for both time scales, we believe that this approach provided deeper insights into the disease-risk association since the additional use of calendar time allows to indirectly account for transmission-associated effects.

## Abbreviations

HR, hazard ratio; ICU, intensive care unit; MRSA, methicillin-resistant staphylococcus aureus; sHR, subdistribution hazard ratio

## Additional file

Additional file 1 Additional plots (Lexis diagram, variation due to different ICUs) and statistical code (in SAS and R) is provided in Additional file 1.pdf. (PDF 111 kb)

## References

[1] Brown KA, Daneman N, Stevens VW, Zhang Y, Greene TH, Samore MH (2016). Integrating time-varying and ecological exposures into multivariate analyses of hospital-acquired infection risk factors: a review and demonstration. Infect Control Hosp Epidemiol.

[2] Bootsma MC, Bonten MJ, Nijssen S, Fluit AC, Diekmann O (2007). An algorithm to estimate the importance of bacterial acquisition routes in hospital settings. Am J Epidemiol.

[3] Cooper BS, Medley GF, Bradley SJ, Scott GM (2008). An augmented data method for the analysis of nosocomial infection data. Am J Epidemiol.

[4] Cheung YB, Gao F, Khoo, KS (2003). Age at diagnosis and the choice of survival analysis methods in cancer epidemiology. J Clin Epidemiol.

[5] Korn EL, Graubard BI, Midthune D (1997). Time-to-event analysis of longitudinal follow-up of a survey: choice of the time-scale. Am J Epidemiol.

[6] Griffin BA, Anderson GL, Shih RA, Whitsel EA (2012). Use of alternative time scales in Cox proportional hazard models: implications for time-varying environmental exposures. Stat Med.

[7] Arjas E, Keiding N, Borgan Ø, Andersen PK, Natvig B (1989). Survival models and martingale dynamics [with Discussion and Reply]. Scand J Stat.

[8] Vandenbroucke JP, Pearce N (2012). Incidence rates in dynamic populations. Int J Epidemiol.

[9] Wolkewitz M, Dettenkofer M, Bertz H, Schumacher M, Huebner J (2008). Statistical epidemic modeling with hospital outbreak data. Stat Med.

[10] Gastmeier P, Stamm-Balderjahn S, Hansen S, Zuschneid I, Groneberg K (2005). How outbreaks can contribute to prevention of nosocomial infection: analysis of 1,022 outbreaks. Infect Control Hosp Epidemiol.

[11] Albrich WC, Harbarth S (2008). Health-care workers: source, vector, or victim of MRSA?. Lancet Infect Dis.

[12] Wolkewitz M, Vonberg RP, Grundmann H (2008). Risk factors for the development of nosocomial pneumonia and mortality on intensive care units: application of competing risks models. Crit Care.

[13] Wolkewitz M, Cooper B, Palomar-Martinez M, Alvarez-Lerma F, Olaechea-Astigarraga P, Barnett A (2014). Multilevel competing risk models to evaluate the risk of nosocomial infection. Crit Care.

[14] Wolkewitz M, Cooper BS, Bonten MJ, Barnett AG, Schumacher M (2014). Interpreting and comparing risks in the presence of competing events. BMJ.

[15] Wolkewitz M, Harbarth S, Beyersmann J (2013). Daily chlorhexidine bathing and hospital-acquired infection. N Engl J Med.

[16] Schumacher M, Allignol A, Beyersmann J, Binder N, Wolkewitz M (2013). Hospital-acquired infections: appropriate statistical treatment is urgently needed. Int J Epidemiol.

[17] Andersen PK, Geskus RB, de Witte T, Putter H (2012). Competing risks in epidemiology: possibilities and pitfalls. Int J Epidemiol.

[18] Wolkewitz M. Accounting for competing events in multivariate analyses of hospital-acquired infection risk factors. Infect Control Hospital Epidemiol. (in press). 10.1017/ice.2016.162 27452870

[19] Latouche A, Allignol A, Beyersmann J, Labopin M, Fine JP (2013). A competing risks analysis should report results on all cause-specific hazards and cumulative incidence functions. J Clin Epidemiol.

[20] Lopez-Pueyo MJ, Olaechea-Astigarraga P, Palomar-Martinez M, Insausti-Ordenana J, Alvarez-Lerma F, Group EHS (2013). Quality control of the surveillance programme of ICU-acquired infection (ENVIN-HELICS registry) in Spain. J Hosp Inf.

[21] Plummer M, Carstensen B (2011). Lexis: An R class for epidemiological studies with long-term follow-up. J Stat Softw.

[22] Rondeau V, Mazroui Y, Gonzalez JR (2012). frailtypack: An R package for the analysis of correlated survival data with frailty models using penalized likelihood estimation or parametrical estimation. J Stat Softw.

[23] Fine J, Gray RJ (1999). A proportional hazards model for the subdistribution of a competing risk. J Am Stat Assoc.

[24] Schoenfeld D (1982). Partial residuals for the proportional hazards regression model. Biometrika.

[25] Grambauer N, Schumacher M, Beyersmann J (2010). Proportional subdistribution hazards modeling offers a summary analysis, even if misspecified. Stat Med.

[26] Andersen PK, Abildstrom SZ, Rostha Sj (2002). Competing risks as a multi-state model. Stat Methods Med Res.

[27] Lau B, Cole SR, Gange SJ (2009). Competing risk regression models for epidemiologic data. Am J Epidemiol.

[28] Palomar M, Alvarez-Lerma F, Riera A, Diaz MT, Torres F, Agra Y (2013). Impact of a national multimodal intervention to prevent catheter-related bloodstream infection in the ICU: the Spanish experience. Crit Care Med.

[29] Andersen PK, Keiding N (2006). Survival and event history analysis.

[30] Pencina MJ, Larson MG, D’Agostino RB (2007). Choice of time scale and its effect on significance of predictors in longitudinal studies. Stat Med.

