# Supplementary material for: Multiple time scales in modeling the incidence of infections acquired in intensive care units
Source: BMC Med Res Methodol. 2016 Sep 1;16(1):116. doi: 10.1186/s12874-016-0199-y (PMC5009530; doi:10.1186/s12874-016-0199-y)
Supplement: Additional file 1 — Additional plots (Lexis diagram, variation due to different ICUs) and statistical code (in SAS and R) is provided in Additional file 1.pdf. (PDF 111 kb) [file 12874_2016_199_MOESM1_ESM.pdf]

Multiple time scales in modeling the incidence of infections acquired in intensive care units

- APPENDIX -

- Additional plots
- Statistical code

Figure 1: Lexis diagram of one selected ICU over 100 calendar days. Individual patients are displayed in both ICU and calendar time until he or she acquired a nosocomial MRSA infection (marked with an 'o'), died without MRSA (marked with an '+') or is discharged without MRSA (no marking).

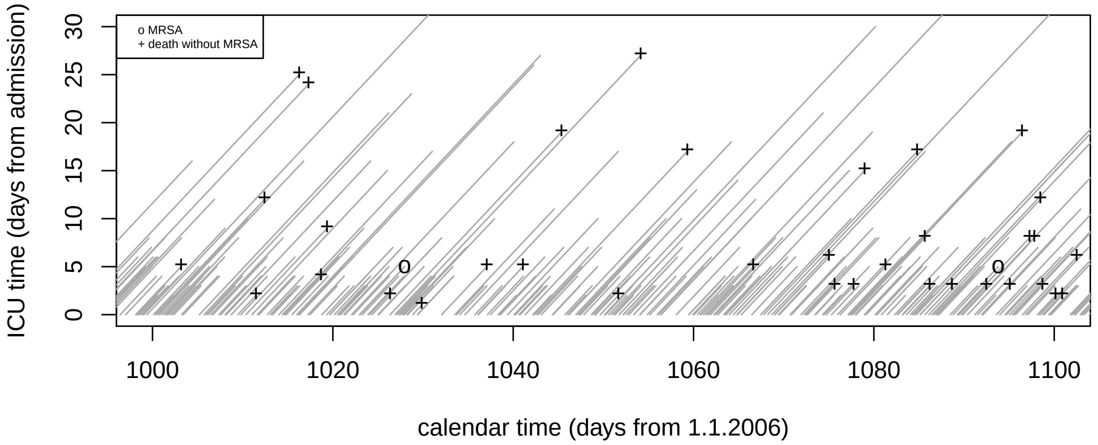

Figure 2: Variation due to different ICUs: estimated frailties for each corresponding event. The upper panels shows the frailties when ICU time is used as the basic time scale and the lower panels shows the frailties when calendar time is used. The variation is larger for MRSA infections (θ=0.384 (SE 0.097)) than for the competing events (θ=0.110 (SE 0.019) for death and θ=0.087 (SE 0.014) for discharge); a pattern which has been described more generally for nosocomial bacteraemia using the same data base (Wolkewitz et al. Crit Care, 2014; 18 (online): R64.). When switching the time scale to calendar time (lower figures), the variations remain almost the same (θ= 0.39 (SE 0.098) for MRSA, 0.105 (SE 0.018) for death and θ=0.122 (SE 0.019) for discharge without MRSA).

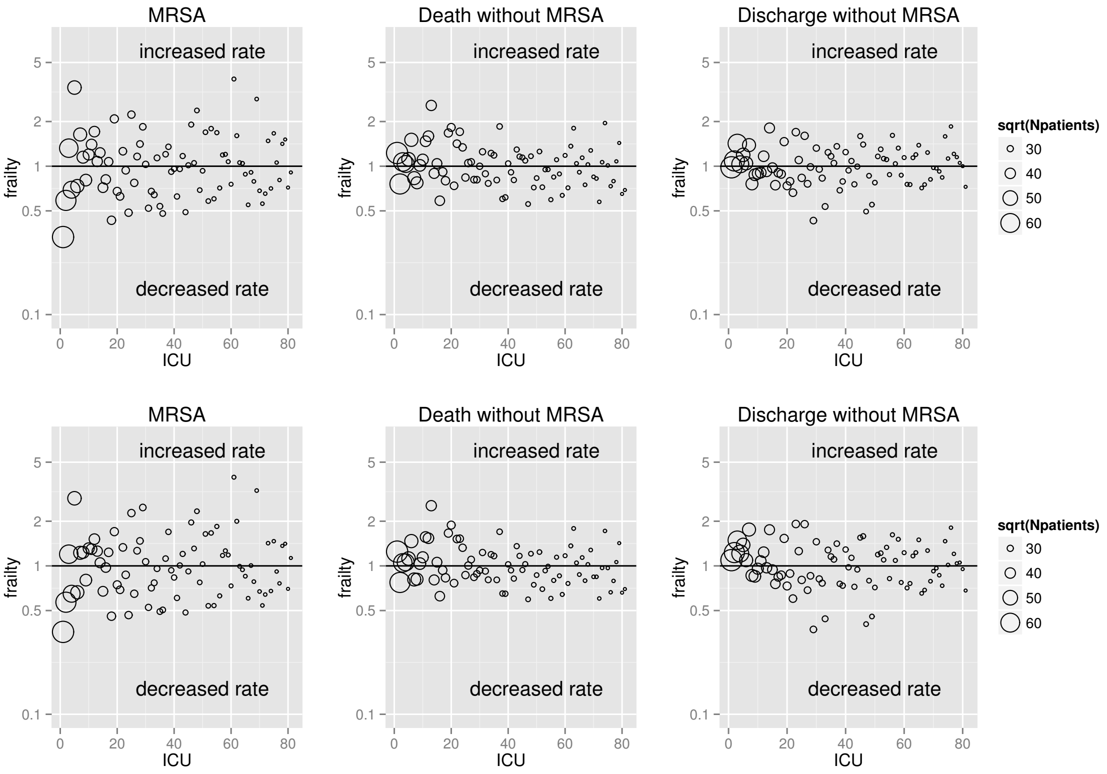

Statistical code in SAS

Estimating the Frailty parameter Theta for each outcome and corresponding time scale, shared frailty model (patients within ICU share the same frailty)

```
 * to=1 is MRSA, to=2 is death, to=3 is discharge and to=4 is censored;
 * stop is the ICU time of mrsa, death, discharge or censored whatever comes first;
 * start_c and stop_c are the calendar difference times from 1.1.2006;

 * ICU time scale, outcome: MRSA;
proc phreg  data=mrsa;
  model stop*to(2,3,4)=  / rl;
  random hospital_id / solution;
run;

* ICU time scale, outcome: death without MRSA;
proc phreg  data=mrsa;
  model stop*to(1,3,4)=  / rl;
  random hospital_id / solution;
run;

* ICU time scale, outcome: discharge without MRSA;
proc phreg  data=mrsa;
  model stop*to(1,2,4)=  / rl;
  random hospital_id / solution;
run;

* calendar time scale, outcome: MRSA;
proc phreg  data=mrsa;
  model (start_c,stop_c)*to(2,3,4)=  / rl;
  random hospital_id / solution;
run;

* calendar time scale, outcome: death without MRSA;
proc phreg  data=mrsa;
  model (start_c,stop_c)*to(1,3,4)=  / rl;
  random hospital_id / solution;
run;

* calendar time scale, outcome: discharge without MRSA;
proc phreg  data=mrsa;
  model (start_c,stop_c)*to(1,2,4)=  / rl;
  random hospital_id / solution;
run;
```

Multivariate analysis for each outcome and corresponding time scale, stratified for hospital ID

```
 * ICU time scale, outcome: MRSA;
proc phreg  data=mrsa;
  class hospital_id my_diag year_in_c apache_c age_c time_hosp_before_icu_c
     / ref=first;
  model stop*to(2,3,4)=  year_in_c apache_c age_c
    time_hosp_before_icu_c my_diag my_atb48h my_sex my_origen my_trauma / rl;
  strata hospital_id ;
run;

* ICU time scale, outcome: death without MRSA;
proc phreg  data=mrsa;
  class hospital_id my_diag year_in_c apache_c age_c time_hosp_before_icu_c
     / ref=first;
  model stop*to(1,3,4)=  year_in_c apache_c age_c
    time_hosp_before_icu_c my_diag my_atb48h my_sex my_origen my_trauma / rl;
  strata hospital_id ;
run;

* ICU time scale, outcome: discharge without MRSA;
proc phreg  data=mrsa;
  class hospital_id my_diag year_in_c apache_c age_c time_hosp_before_icu_c
     / ref=first;
  model stop*to(1,2,4)= year_in_c apache_c age_c
    time_hosp_before_icu_c my_diag my_atb48h my_sex my_origen my_trauma / rl;
  strata hospital_id ;
run;

* calendar time scale, outcome: MRSA;
proc phreg  data=mrsa;
  class hospital_id my_diag year_in_c apache_c age_c time_hosp_before_icu_c
     / ref=first;
  model (start_c,stop_c)*to(2,3,4)= year_in_c apache_c age_c
    time_hosp_before_icu_c my_diag my_atb48h my_sex my_origen my_trauma / rl;
  strata hospital_id ;
run;

* calendar time scale, outcome: death without MRSA;
proc phreg  data=mrsa;
  class hospital_id my_diag year_in_c apache_c age_c time_hosp_before_icu_c
     / ref=first;
  model (start_c,stop_c)*to(1,3,4)= year_in_c apache_c age_c
    time_hosp_before_icu_c my_diag my_atb48h my_sex my_origen my_trauma / rl;
  strata hospital_id ;
run;

* calendar time scale, outcome: discharge without MRSA;
proc phreg  data=mrsa;
  class hospital_id my_diag year_in_c apache_c age_c time_hosp_before_icu_c
     / ref=first;
  model (start_c,stop_c)*to(1,2,4)= year_in_c apache_c age_c
    time_hosp_before_icu_c my_diag my_atb48h my_sex my_origen my_trauma / rl;
  strata hospital_id ;
run;
```

Hazard plots (R code)

```
library("frailtypack")

# icu time
inf<-frailtyPenal(Surv(stop,to==1) ~ cluster(hospital_id) ,
                  data=blow_,n.knots=10,kappa=1720498,
                  Frailty=TRUE,cross.validation=TRUE)

dea<-frailtyPenal(Surv(stop,to==2) ~ cluster(hospital_id) ,
                  data=blow_,n.knots=10,kappa1=160465447,
                  Frailty=TRUE,cross.validation=TRUE)

dis<-frailtyPenal(Surv(stop,to==3) ~ cluster(hospital_id) ,
                  data=blow_,n.knots=10,kappa1=160465447,
                  Frailty=TRUE,cross.validation=TRUE)

# calendar time
infc<-frailtyPenal(Surv(start_c,stop_c,to==1) ~ cluster(hospital_id) ,
                  data=blow_,n.knots=10,kappa1=1720498,
                  Frailty=TRUE,cross.validation=TRUE)

deac<-frailtyPenal(Surv(start_c,stop_c,to==2) ~ cluster(hospital_id) ,
                  data=blow_,n.knots=7,kappa1=160465447,
                  Frailty=TRUE,cross.validation=TRUE)

disc<-frailtyPenal(Surv(start_c,stop_c,to==3) ~ cluster(hospital_id) ,
                  data=blow_,n.knots=10,kappa1=160465447,
                  Frailty=TRUE,cross.validation=TRUE)


# plot hazards
postscript(file="/home/wolke/tex/paper/Scale/hazards_mrsa.eps",width=6, height=4)
par(mfrow=c(2,3))
plot(inf,xlim=c(0,40),main='MRSA',ylim=c(0,0.002))
plot(dea,xlim=c(0,40),main='Death without MRSA',ylim=c(0,0.03))
plot(dis,xlim=c(0,40),main='Discharge without MRSA',ylim=c(0,0.3))
plot(infc,ylim=c(0,0.002))
plot(deac,ylim=c(0,0.03))
plot(disc,ylim=c(0,0.3))
dev.off()
```
